# *Lactobacillus rhamnosus* HN001 and *Lactobacillus acidophilus* La-14 Attenuate *Gardnerella vaginalis*-Infected Bacterial Vaginosis in Mice

**DOI:** 10.3390/nu9060531

**Published:** 2017-05-23

**Authors:** Se-Eun Jang, Jin-Ju Jeong, Su-Young Choi, Hyunji Kim, Myung Joo Han, Dong-Hyun Kim

**Affiliations:** 1 Department of Life and Nanopharmaceutical Sciences, College of pharmacy, Kyung Hee University, 26, Kyungheedae-ro, Dongdaemun-gu, Seoul 02447, Korea; jse3507@naver.com (S.-E.J.); bongoori7@naver.com (J.-J.J.); 2 Department of Food and Nutrition, Kyung Hee University, Seoul 02447, Korea; mjhan@khu.ac.kr; 3 NutriScience Co., Ltd., Seoul 06132, Korea; sychoifwp@nutribiotech.co.kr (S.-Y.C.); hyunji.kim@nutribiotech.co.kr (H.K.)

**Keywords:** bacterial vaginosis, *Lactobacillus rhamnosus*, *Lactobacillus acidophilus*, *Gardnerella vaginalis*

## Abstract

Oral administration of a probiotic mixture (PM; Respecta^®^) consisting of *Lactobacillus rhamnosus* HN001 (L1), *Lactobacillus acidophilus* La-14 (L2), and lactoferrin RCXTM results in colonization of these probiotics in the vagina of healthy women. Therefore, we examined whether vaginal colonization of the PM ingredients L1 and L2 could attenuate bacterial vaginosis (BV). BV was induced in mice via β-estradiol-3-benzoate-induced immunosuppression and intravaginal inoculation with *Gardnerella vaginalis* (GV). Inflammatory markers were analyzed using enzyme-linked immunosorbent assay, immunoblotting, quantitative polymerase chain reaction, and flow cytometry. Oral or intravaginal administration of PM resulted in colonization of L1 and L2 in the vagina. Oral or intravaginal administration of L1, L2, or PM significantly inhibited GV-induced epithelial cell disruption, myeloperoxidase activity, NF-κB activation, and IL-1β and TNF-α expression (*p* < 0.05). Administration of these probiotics also inhibited IL-17 and RORγt expression but increased IL-10 and Foxp3 expression. Of these probiotics, L2 most effectively attenuated GV-induced BV, followed by L1 and PM. Oral administration was more effective against GV-induced BV than intravaginal administration. L1 and L2 also significantly inhibited the adherence of GV to HeLa cells (a human cervical cancer cell line) and GV growth in vitro. In addition, L1 and L2 inhibited lipopolysaccharide-induced NF-κB activation in macrophages and the differentiation of splenocytes into Th17 cells in vitro, but increased their differentiation into Treg cells. Our study suggests that L1, L2, and PM attenuated GV-induced vaginosis by regulating both vaginal and systemic innate and adaptive immune responses rather than direct competition or killing of GV in the vagina.

## 1. Introduction

The vaginal microbiota, which is dominated by lactobacilli, plays an important role in maintaining female health [1,2]. Disturbance of the vaginal microbiota allows infection by various pathogens such as *Gardnerella vaginalis* (GV) and *Atopobium vaginae* (AV), resulting in bacterial vaginosis (BV) [3,4]. BV is a common vaginal inflammatory disease in women that is manifested by malodorous discharge and elevated pH [5,6]. Although antimicrobial drugs, such as clindamycin and metronidazole, are recommended for the treatment of BV, the use of these drugs is limited due to their side effects such as drug resistance and superinfection [7,8]. Therefore, probiotics are frequently used to treat BV [9,10]. 

Lactobacilli are safe probiotics that antagonize pathogens, have anti-inflammatory (anticolitic and antivaginitic) effects [11,12,13,14], and modulate host immunity. It has been shown that oral or vaginal administration of *Lactobacillus johnsonii* HY7042 attenuated GV-infected BV in mice by inhibiting GV growth and host inflammatory responses [11]. In addition, oral or vaginal administration of a *Lactobacilli* mixture, such as *Lactobacillus rhamnosus* GR-1 and *Lactobacillus fermentum* RC-14, which produces hydrogen peroxide and bacteriocins, suppressed the recurrence of BV [15,16]. Strus et al. reported that an orally administered probiotic mixture of *Lactobacillus fermentum* 57A, *Lactobacillus plantarum* 57B, and *Lactobacillus gasseri* 57C colonized the vagina of healthy women [17]. Arberti et al. also reported that oral administration of a probiotic mixture (PM; Respecta^®^) consisting of *Lactobacillus rhamnosus* HN001 (L1), *Lactobacillus acidophilus* La-14 (L2), and lactoferrin RCXTM resulted in colonization of the probiotics in the vaginas of healthy women [18]. These results suggest that orally administered probiotics may ameliorate BV by colonizing the vagina. However, there are few studies on the relationship between probiotic colonization and the BV-curative effect of these probiotics. 

Therefore, to understand the relationship between the vaginal colonization and anti-GV activity of probiotics, we examined the effects of L1 and L2 alone as well as their mixture (PM) against GV-induced BV in mice.

## 2. Materials and Methods

### 2.1. Materials

Brain heart infusion (BHI) and de Man, Rogosa and Sharpe (MRS) media was purchased from Becton, Dickinson and Company (Sparks, MD, USA). A general anaerobic medium (GAM) broth was purchased from Nissui Pharmaceutical Inc. (Tokyo, Japan). Lipopolysaccharide (LPS) purified from *Escherichia coli* O111:B4, sodium thioglycolate, hydrogen peroxide, β-estradiol-3-benzoate, hematoxylin-eosin, tetramethyl benzidine, DMEM, and RPMI1640 were purchased from Sigma (St. Louis, MO, USA). Enzyme-linked immunosorbent assay (ELISA) kits for cytokines TNF-α, IL-1β, IL-10, and IL-17A were supplied from R&D Systems (Minneapolis, MN, USA). Antibodies from p65, p-65, tumor necrosis factor (TNF)-α, myeloperoxidase were purchased from Cell Signaling Technology (Beverly, MA, USA). Fetal bovine serum (FBS) and heat-inactivated fetal calf serum (FCS) purchased from Panbiotech GmbH (Aidenbach, Germany). A SYBR premix agent was purchased from TaKaRa (Shiga, Japan). Pan T Cell Isolation Kit II was purchased from MiltenyiBiotec GmbH (Bergisch Gladbach, Germany). Anti-CD28, anti-CD3, recombinant IL-6, and recombinant TGF-β were purchased from BioGems International Inc. (Westlake Village, CA, USA). 3,3-Diaminobenzidine substrate kit was purchased from Vector Laboratories, Inc. (Burlingame, CA, USA). Radio immunoprecipitation assay (RIPA) lysis buffer, paraformaldehyde, and phosphate buffered saline (PBS) were purchased from Biosesang (Seoul, Korea). RNeasy Mini Kit was purchased from Qiagen (Hilden, Germany). Respecta^®^, which was consisted of L1 (0.32 × 10^11^ CFU/g), L2 (1.28 × 10^11^ CFU/g), and lactoferrin RCXTM (418 mg/g), was donated from Nutribioscience (Seoul, Korea).

### 2.2. Bacterial Culture

*Gardnerella vaginalis* KCTC5096 (GV) and *Atopobium vaginae* KCTC15240 (AV) were obtained from the Korean Collection for Type Cultures (Daejun, Korea). The bacteria were subcultured in GAM or BHI broth supplemented with yeast extract (1%), maltose (0.1%), glucose (0.1%), and horse serum (10%) at 37 °C for 48 h under anaerobic conditions (in a sealed anaerobic jar). L1 and L2 were cultured under anaerobic conditions in MRS broth at 37 °C for up to 24 h. Cells were harvested and suspended in sterilized PBS to a density of 3 × 10^8^ cells/mL for vaginal inoculation and in vitro experiments.

### 2.3. Assay for the Inhibitory Effects of Probiotics Against the Growth of GV and AV

GV or AV (1 × 10^7^ CFU/mL) was inoculated into 5 mL of GAM broth in the presence or absence of L1 (at 1 × 10^6^, 1 × 10^7^, or 1 × 10^8^ CFU/mL) or L2 (at 1 × 10^6^, 1 × 10^7^, or 1 × 10^8^ CFU/mL) and incubated anaerobically at 37 °C for 24 h. qPCR was performed to detect GV or AV. 

qPCR was performed with 50 ng of total DNA isolated from vaginal fluid or cultured cells with SYBER premix in a Takara thermal cycler [19]. The thermal cycling conditions were as follows: 95 °C for 30 s, followed by 35 cycles of denaturation and amplification at 95 °C for 5 s and 63 °C for 30 s, respectively. Gene expression levels were calculated relative to bacterial rRNA, using Microsoft Excel. 

Primers (Macrogen, Seoul, Korea) were prepared according to the method of de Alberti et al. [18]: L1 forward: 5′-TGC TTG CAT CTT GAT TTA ATT TTG-3′, reverse: 5′-GGT TCT TGG ATY TAT GCG GTA TTA G-3′; L2 forward: 5′-TGC AAA GTG GTA GCG TAA GC-3′, reverse: 5′-CCT TTC CCT CAC GGT ACT G-3′; GV forward: 5′-TTA CTG GTG TAT CAC TGT AAG G-3′, reverse: 5′-CCG TCA CAG GCT GAA CAG T-3′; AV forward: 5′-GGT GAA GCA GTG GAA ACA CT-3′, reverse: 5′-ATT CGC TTC TGC TCG CGC A-3′; 16S rDNA forward: 5′-AGA GTT TGA TCC TGG CTC AG-3′, reverse: 5′-AAG GAG GTG WTC CAR CC-3′.

In the preliminary study, we confirmed whether tested bacteria could be detectable: GV, AV, L1, or L2 (1 × 10^3^, 1 × 10^5^, 1 × 10^7^, or 1 × 10^9^ CFU/mL) was supplemented in saline or vaginal tissue homogenate (10 mg/mL), DNA was extracted, and qPCR for each bacterium was performed.

### 2.4. Assay for the Antagonistic Effects of Probiotics on the Adherence of GV to HeLa Cells

The antagonistic effects of the probiotics on the adherence of GV to HeLa cells were assayed according to the method of Joo et al. [9]. HeLa cells (KCLB10002; Korea Cell Line Bank, Seoul, Korea) were cultured in 5% CO_2_/95% air in RPMI 1640 supplemented with 10% heat-inactivated FCS at 37 °C and then washed twice with DMEM. HeLa cells (1 × 10^8^ cells/mL in 0.5 mL of DMEM) were incubated in 24-well culture plates for 1 h, and then incubated with GV (1 × 10^7^ CFU/mL, suspended in DMEM) or vehicle for 1 h in the absence or presence of L1 or L2 (at 1 × 10^5^, 1 × 10^6^, 1 × 10^7^ CFU/mL, suspended in DMEM) at 37 °C for 24 h. The plates were washed three times with DMEM, and the numbers of L1, L2, and GV attached to the cells were assayed using qPCR. 

### 2.5. Animals

Seven-week-old female C57BL/6 mice weighing 19–22 g were obtained from Koatech Co. (Gyeonggi, Korea). The mice were housed in wire cages under climate-controlled conditions (at 50 ± 10% humidity and 20–22 °C), fed standard laboratory chow, and allowed water ad libitum. The animal experiments were approved by the Committee for the Care and Use of Laboratory Animals in the Kyung Hee University (IRB No. KHUASP(SE)-16-126), and all animal handling was performed in accordance with the Guidelines for the Care and Use of Laboratory Animals of Kyung Hee University.

### 2.6. Preparation of Macrophages

Macrophages were removed from the peritoneal cavity of mice by intraperitoneal injection with 2 mL of 4% (*w*/*v*) thioglycolate, and the obtained cells were washed twice with RPMI 1640 [14]. Then, the cells (1.5 × 10^6^ cells/well) were incubated in RPMI 1640 containing 1% antibiotic-antimycotic and 10% FBS at 37 °C for 20 h, and washed three times. Attached cells were used as macrophages.

Macrophages (1 × 10^6^ cells/well) were treated with LPS (100 ng/mL) in the absence or presence of probiotics (at 1 × 10^3^, 1 × 10^4^, or 1 × 10^5^ CFU/mL) for 90 min (for p65 and p-p65 [NF-κB]) or 20 h (for TNF-α).

### 2.7. Preparation of Splenocytes

Mouse spleens were aseptically removed, gently crushed, and lysed in tris-buffered ammonium chloride (Biosesang, Seoul, Korea) according to the method of Lim et al. [20]. Cells were suspended in RPMI 1640 medium containing 10% FCS and T cells were isolated by magnetic cell sorting with the Pan T Cell Isolation Kit II. 

For differentiation into helper T17 (Th17) cells, the purified T cells (1 × 10^7^ cells/well) were stimulated with anti-CD28 (1 μg/mL) and anti-CD3 (1 μg/mL) antibodies, recombinant IL-6 (20 ng/mL), and recombinant TGF-β (1 ng/mL) for 5 days. For differentiation into regulatory T (Treg) cells, purified Th cells (0.3 × 10^7^ cells/well) were stimulated with anti-CD3 (1 μg/mL) and anti-CD28 (1 μg/mL) antibodies for 5 days. The differentiated T cells were fixed and stained with anti-CD4, anti-CD25, anti-FoxP3, or anti-IL-17A antibodies and then analyzed by flow cytometry. 

### 2.8. Induction of BV in Mice 

**Figure 1:**
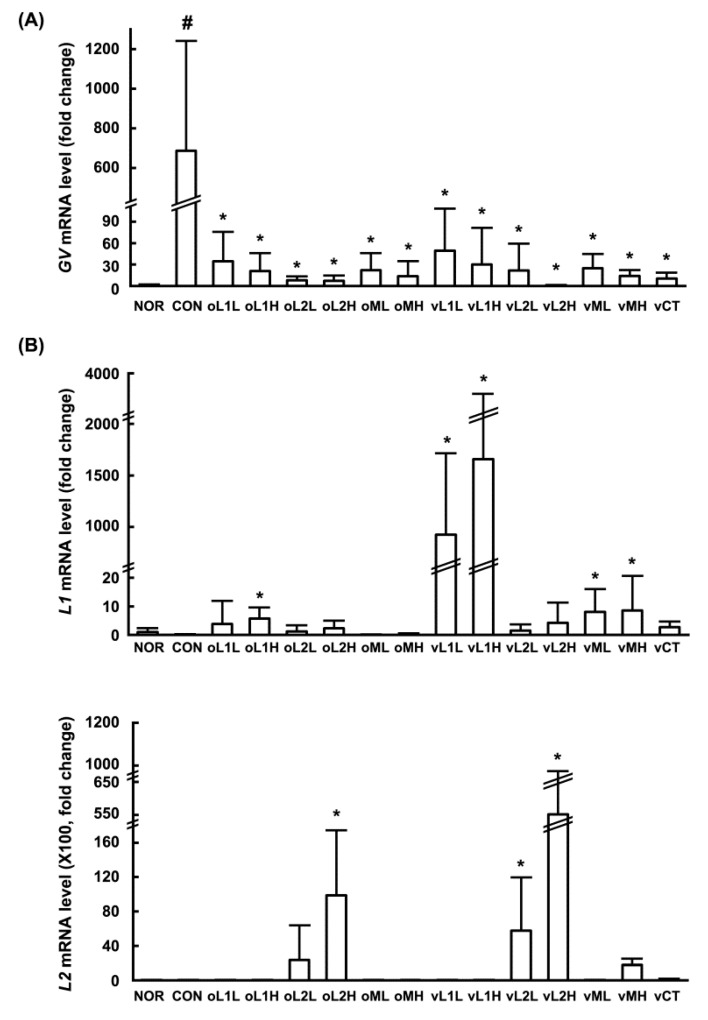
Effects of *Lactobacillus rhamnosus* HN001 (L1), *Lactobacillus acidophilus* La-14 (L2), and PM in mice against the vaginal infection of *Gardnerella vaginalis* (GV). (**A**) Effects on the number of GV in the vaginal tissue; (**B**) Effects on the numbers of L1 and L2 in the vaginal tissues. Female mouse vaginas were infected with *G. vaginalis* (6 × 10^6^ CFU/mouse) except normal group (NOR, normal group treated with vehicle alone). Test agents (CON, vehicle alone; oL1L, orally administered L1; oL1H, orally administered 5 × 10^9^ CFU/mouse of L1; oL2L, orally administered 5 × 10^8^ CFU/mouse of L2; oLr2H, orally administered 5 × 10^9^ CFU/mouse of L2; oML, orally administered 5 × 10^8^ CFU of PM/mouse; oMH, orally administered 5 × 10^9^ CFU of PM/mouse; vL1L, intravaginally administered 5 × 10^8^ CFU/mouse of L1; vL1H, intravaginally administered 5 × 10^9^ CFU/mouse of L1; vL2L, intravaginally administered 5 × 10^8^ CFU/mouse of L2; vLr2H, intravaginally administered 5 × 10^9^ CFU/mouse of L2; vML, intravaginally administered 5 × 10^8^ CFU of PM/mouse; vMH, intravaginally administered 5 × 10^9^ CFU of PM/mouse; and vCT, intravaginally administered 20 μL of 10% (*v/v*) clotrimazole) were administered once a day for 14 days. On day 15 post-infection, the mice were sacrificed. The number of GV, L2, and L1 were measured in the vaginal cavity using qPCR. All values are shown as the mean ± SD (*n* = 6). ^#^ *p* < 0.05 vs. normal control group. * *p* < 0.05 vs. GV-treated control group.

BV was induced according to the method of Joo et al. [11]. Mice were acclimatized for 7 days before starting the experiments and were separated into 15 groups of six mice each. β-Estradiol-3-benzoate (0.5 mg/0.1 mL) was subcutaneously injected into all mice, except in the normal control group, 72 h before GV infection, and then a suspension of GV (1 × 10^6^ CFU /20 μL saline) was administered intravaginally. The normal control group (NOR) was treated with saline instead of the GV suspension. Probiotics were administered either orally or intravaginally once a day for 14 days beginning the day after infection (Figure 1). The GV-infected control group (CON) was treated with saline (vehicle) instead of probiotics. Mice were sacrificed 24 h after the final probiotic treatment. Vaginas were washed with PBS, and DNA was isolated with the DNeasy Blood & Tissue Kit. The washed and excised vaginas were stored at −80 °C for myeloperoxidase activity assay, immunoblotting, ELISA, and qPCR or overnight post-fixed in 50 mM phosphate buffer (pH 7.4) containing 4% paraformaldehyde for histological examination.

### 2.9. Histopathological Examination

Fixed tissues were dehydrated in methanol, sectioned (20-μm thick), and stained with hematoxylin-eosin. The tissues were also immunostained with anti-myeloperoxidase or TNF-α antibody and the 3,3-diaminobenzidine substrate kit and visualized with 3-amino-9-ethylcarbazole.

### 2.10. Myeloperoxidase Activity Assay

Vaginal tissues were homogenized in RIPA lysis buffer (300 µL; Biosesang, Seoul, Korea) containing 1% phosphatase and 1% protease inhibitor cocktail and centrifuged at 10,000× *g*, for 20 min at 4 °C [14]. The supernatant (50 µL) was added to a reaction mixture containing 1.6 mM tetramethyl benzidine and 0.1 mM hydrogen peroxide, incubated at 37 °C and then the absorbance at 650 nm was measured over time. 

### 2.11. ELISA and Immunoblotting

For cytokine analysis by ELISA, the supernatant from vaginal tissue homogenates was transferred to 96-well ELISA plates, and the concentrations of TNF-α, IL-1β, IL-10, and IL-17A were measured using ELISA kits (R&D Systems). 

For immunoblotting, the proteins in the supernatant from vaginal tissue homogenates were separated by sodium dodecyl sulfate-polyacrylamide gel electrophoresis. Then, the separated proteins were transferred to a nitrocellulose membrane (Sigma) and immunodetected according to the method of Jang et al. [14].

### 2.12. Quantitative Polymerase Chain Reaction (qPCR)

Real-time PCR analysis of IL-10, RAR-related orphan receptor gamma t (RORγt), Foxp3, and glyceraldehyde 3-phosphate dehydrogenase (GAPDH) was performed according to the method of Lim et al. [20]. Total RNA was isolated from the vagina using the RNeasy Mini Kit, and cDNA was prepared from 2 μg of purified RNA using Takara reagents. qPCR was performed with SYBER premix in a Qiagen thermal cycler. The thermal cycling conditions were as follows: 95 °C for 5 min, followed by 36 cycles of denaturation and amplification at 95 °C for 10 s and 60 °C for 30 s, respectively. Gene levels were calculated relative to GAPDH using Microsoft Excel. Primers were used as follows: RORγt forward: 5′-ACA GCC ACT GCA TTC CCA GTTT-3′, reverse: 5′-TCT CGG AAG GAC TTG CAG ACAT-3′; Foxp3 forward: 5′-CCC ATC CCC AGG AGT CTT-3′, reverse: 5′-ACC ATG ACT AGG GGC ACT GTA-3′; and GAPDH forward: 5′-TGC AGT GGC AAA GTG GAG AT-3′, reverse: 5′-TTT GCC GTG AGT GGA GTC AT-3′.

### 2.13. Statistical Analysis

All data were indicated as the mean ± standard deviation, with statistical significance analyzed using one-way ANOVA followed by post hoc analysis—Dunnett’s comparison tests. *p* values of 0.05 or less were considered statistically significant.

## 3. Results

### 3.1. Effects of PM, L1, and L2 on BV in Mice

Oral administration of the probiotic mixture (PM; containing L1 and L2) resulted in the colonization of L1 and L2 in the vaginas of healthy women [18]. To determine whether these probiotics could also colonize the vaginas of mice, we orally or intravaginally administered PM or one of its probiotic components, L1 or L2, and assessed the vaginas from the presence of attached probiotics by qPCR (Figure 1). L1 and L2 were detected in the vaginas of mice that were orally or intravaginally administered PM, and more L2 was detected than L1. The levels of L1 and L2 were higher in intravaginally administered mice than in orally administered mice. When L1 or L2, one of the PM ingredients, was orally or intravaginally administerd individually, more L2 was detected than L1.

**Figure 2:**
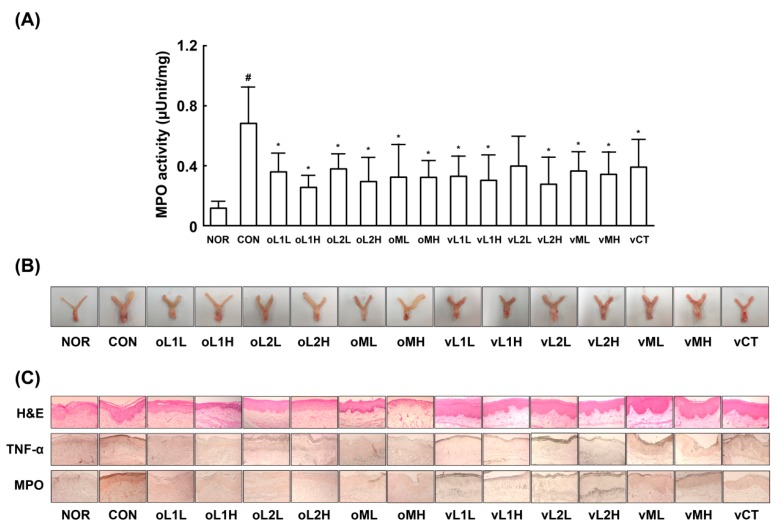
Effects of *Lactobacillus rhamnosus* HN001 (L1), *Lactobacillus acidophilus* La14 (L2), and PM on *Gardnerella vaginalis* (GV)-induced vaginosis in immunosuppressed mice. (**A**) Effects on myeloperoxidase activity in vaginal tissue; (**B**) Effects on GV-inflamed vagina and uterus; (**C**) Histological examination of vaginal tissues, stained with hematoxylin-eosin (upper) and immunostained with anti-myeloperoxidase (middle) or anti-TNF-α antibody (bottom). Female mouse vaginas were infected with GV (1 × 10^8^ CFU/mouse) except normal control group (NOR, normal group treated with vehicle alone). Test agents (CON, vehicle alone; oL1L, orally administered L1; oL1H, orally administered 5 × 10^9^ CFU/mouse of L1; oL2L, orally administered 5 × 10^8^ CFU/mouse of L2; oLr2H, orally administered 5 × 10^9^ CFU/mouse of L2; oML, orally administered 5 × 10^8^ CFU of PM /mouse; oMH, orally administered 5 × 10^9^ CFU of PM/mouse; vL1L, intravaginally administered 5 × 10^8^ CFU/mouse of L1; vL1H, intravaginally administered 5 × 10^9^ CFU/mouse of L1; vL2L, intravaginally administered 5 × 10^8^ CFU/mouse of L2; vLr2H, intravaginally administered 5 × 10^9^ CFU/mouse of L2; vML, intravaginally administered 5 × 10^8^ CFU of PM/mouse; vMH, intravaginally administered 5 × 10^9^ CFU of PM/mouse; and vCT, intravaginally administered 20 μL of 10% (*v/v*) clotrimazole) were administered once a day for 14 days. On day 15 post-infection, the mice were sacrificed. Myeloperoxidase activity was measured in the vaginal tissues. All values are shown as the mean ± SD (*n* = 6). ^#^ *p* < 0.05 vs. normal control group. * *p* < 0.05 vs. GV-treated control group.

**Figure 3:**
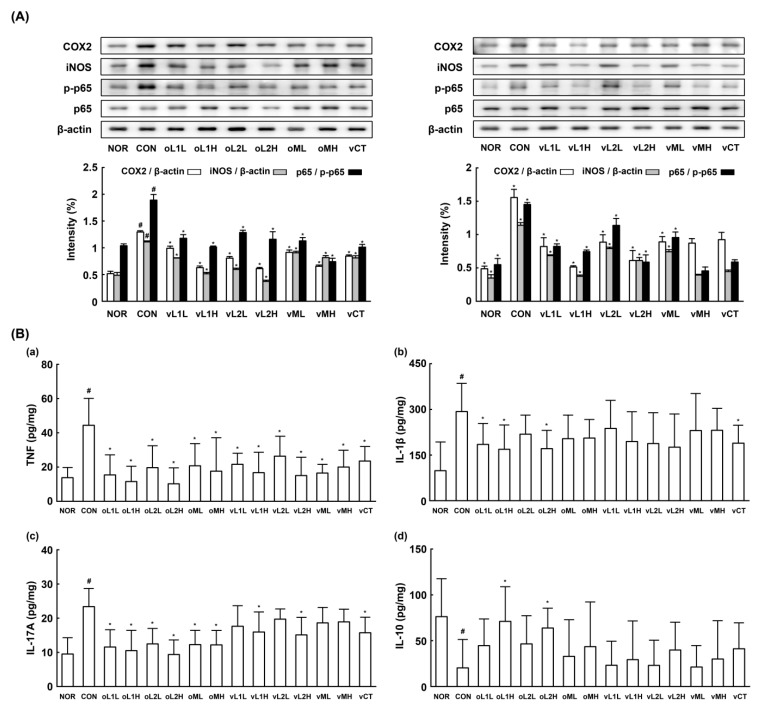
Effects of *Lactobacillus rhamnosus* HN001 (L1), *Lactobacillus acidophilus* La-14 (L2), and PM on the expressions of inflammatory cytokines, COX-2, and iNOS and the activation of NF-κB in the vaginas of *G. vaginalis* (GV)-infected mice. (**A**) Effect in COX-2 and iNOS expressions and NF-κB activation, analyzed by immunoblotting; (**B**) Effects in TNF-α (**a**), IL-1β (**b**), IL-17 (**c**), and IL-10 expressions, assayed by ELISA (**d**). Female mouse vaginas were infected with GV (1 × 10^8^ CFU/mouse) except normal group (NOR, normal group treated with vehicle alone). Test agents (CON, vehicle alone; oL1L, orally administered L1; oL1H, orally administered 5 × 10^9^ CFU/mouse of L1; oL2L, orally administered 5 × 10^8^ CFU/mouse of L2; oLr2H, orally administered 5 × 10^9^ CFU/mouse of L2; oML, orally administered 5 × 10^8^ CFU of PM /mouse; oMH, orally administered 5 × 10^9^ CFU of PM/mouse; vL1L, intravaginally administered 5 × 10^8^ CFU/mouse of L1; vL1H, intravaginally administered 5 × 10^9^ CFU/mouse of L1; vL2L, intravaginally administered 5 × 10^8^ CFU/mouse of L2; vLr2H, intravaginally administered 5 × 10^9^ CFU/mouse of L2; vML, intravaginally administered 5 × 10^8^ CFU of PM/mouse; vMH, intravaginally administered 5 × 10^9^ CFU of PM/mouse; and vCT, intravaginally administered 20 μL of 10% (*v/v*) clotrimazole) were administered once a day for 14 days. On day 15 post-infection, the mice were sacrificed. Inflammatory markers were assayed using ELISA and immunoblot analyses. All values were indicated as mean ± SD (*n* = 6). ^#^ Significantly different vs. normal group (*p* < 0.05). * Significantly different vs. control group (*p* < 0.05).

Next, we investigated whether L1, L2, or PM could attenuate GV-induced vaginosis in mice. GV infection in β-estradiol-immunosuppressed mice caused BV, as evidenced by epithelial cell disruption, increased myeloperoxidase activity (most abundantly expressed in neutrophils recruited to inflammatory tisssues), and upregulation of TNF-α expression (Figure 2). Oral or intravaginal administration of L1, L2, or PM significantly inhibited GV-induced epithelial cell disruption, and myeloperoxidase activity. Vaginal myeloperoxidase activity was dependent on the detected levels of GV. Furthermore, GV infection in the vagina also induced NF-κB activation and iNOS and COX-2 expression. Treatment with L1, L2, or PM significantly inhibited GV-induced NF-κB activation and iNOS and COX-2 expression (*p* < 0.05; Figure 3). Treatment with L1, L2, or PM also inhibited GV-induced IL-1β, IL-17, and TNF-α expression (Figure 3). In contrast, IL-10 expression was increased by treatment with L1, L2, or PM. 

**Figure 4:**
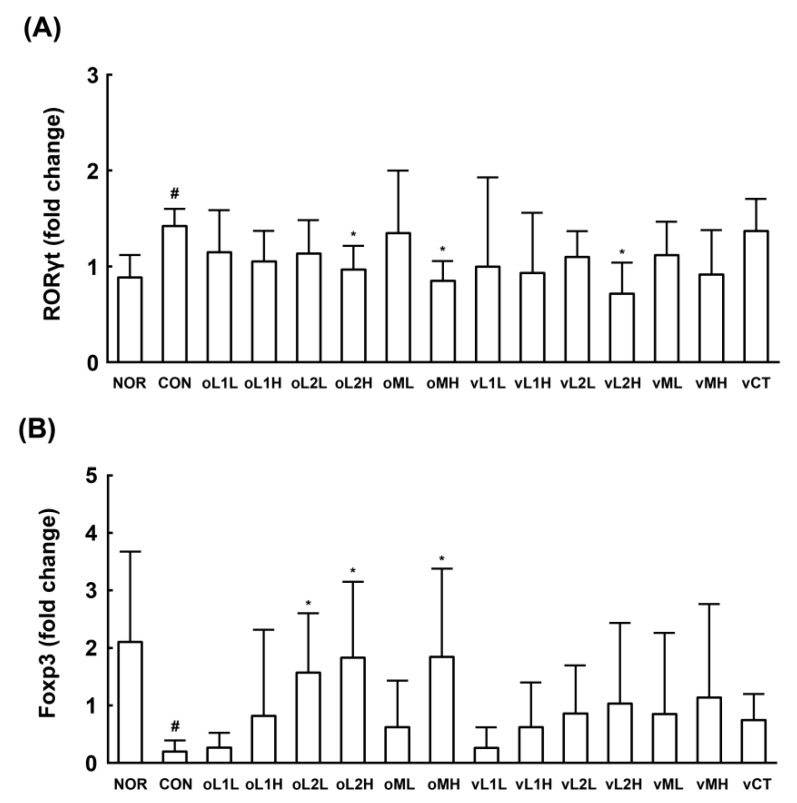
Effects of *Lactobacillus rhamnosus* HN001 (L1), *Lactobacillus acidophilus* La-14 (L2), and PM on the expression of helper T cell transcription factors in the vaginas of immunosuppressed mice. The expression levels of RORγt (**A**) and Foxp3 (**B**) were assessed by qPCR. Test agents (CON, vehicle alone; oL1L, orally administered L1; oL1H, orally administered 5 × 10^9^ CFU/mouse of L1; oL2L, orally administered 5 × 10^8^ CFU/mouse of L2; oLr2H, orally administered 5 × 10^9^ CFU/mouse of L2; oML, orally administered 5 × 10^8^ CFU of PM /mouse; oMH, orally administered 5 × 10^9^ CFU of PM/mouse; vL1L, intravaginally administered 5 × 10^8^ CFU/mouse of L1; vL1H, intravaginally administered 5 × 10^9^ CFU/mouse of L1; vL2L, intravaginally administered 5 × 10^8^ CFU/mouse of L2; vLr2H, intravaginally administered 5 × 10^9^ CFU/mouse of L2; vML, intravaginally administered 5 × 10^8^ CFU of PM/mouse; vMH, intravaginally administered 5 × 10^9^ CFU of PM/mouse; and vCT, intravaginally administered 20 μL of 10% (*v/v*) clotrimazole) were administered once a day for 14 days. All values are shown as the mean ± SD (*n* = 6). ^#^ *p* < 0.05 vs. normal control group. * *p* < 0.05 vs. GV-treated control group.

We also investigated the effect of L1, L2 and PM administration on Th17 and Treg cell differentiation in mice (Figure 4). GV infection in β-estradiol-immunosuppressed mice increased expression of the Th17 transcription factor RORγt in the vagina, as shown by qPCR but suppressed the expression of the Treg transcription factor Foxp3. In addition, oral or intravaginal administration of L1, L2, or PM downregulated GV-induced RORγt expression but upregulated GV-suppressed Foxp3 expression. Overall, the anti-BV effects of orally administered L1, L2, or PM were greater than the effects of intravaginally administered probiotics. Of the tested probiotics, L2 most potently attenuated GV-induced vaginosis, followed by PM and L1.

### 3.2. Growth-Inhibitory Effects of L1 and L2 Against GV

**Figure 5:**
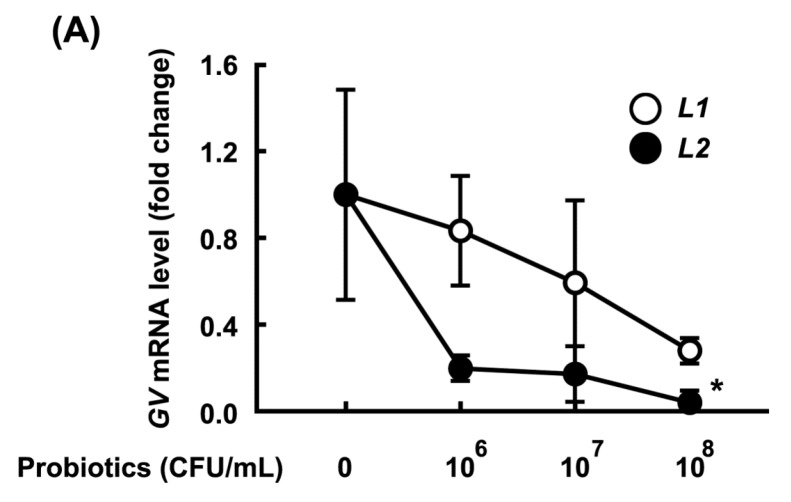

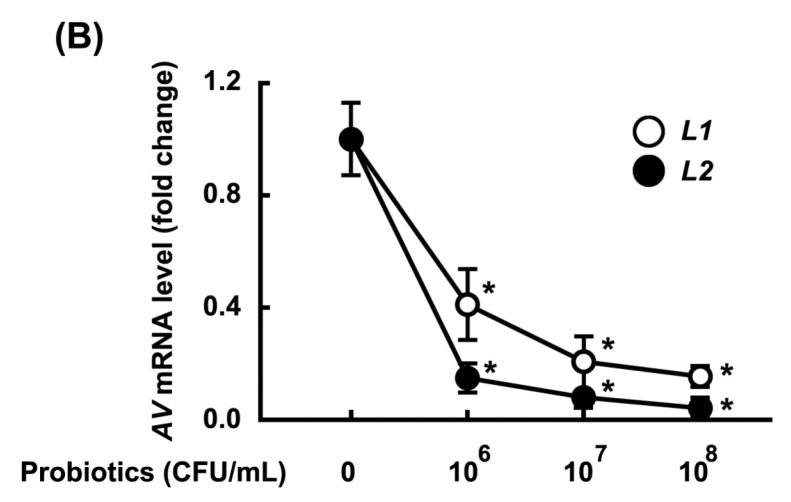
Effects of *Lactobacillus rhamnosus* HN001 (L1) and *Lactobacillus acidophilus* La14 (L2) on the viability of *Gardnerella vaginalis* (**A**) and *Atopobium vaginae* (**B**). The inoculum of *G. vaginalis* (GV) or *A. vaginae* (AV) contained 1 × 10^7^ CFU/mL. The pathogens were incubated without or with L1 (open circle, 1 × 10^6^, 1 × 10^7^, 1 × 10^8^ CFU/mL) or L2 (closed circle, 1 × 10^6^, 1 × 10^7^, 1 × 10^8^ CFU/mL) at 37 °C for 24 h, and number of the survival The numbers of GV and AV were assayed using qPCR. All data are expressed as mean ± SD (*n* = 3). All values are shown as the mean ± SD (*n* = 4). * *p* < 0.05 vs. control group treated with GV or AC alone.

The growth inhibitory activity of L1 and L2 (at 1 × 10^6^, 1 × 10^7^, and 1 × 10^8^ CFU/mL) against GV or AV (1 × 10^8^ CFU/mL) was measured (Figure 5). L1 and L2 potently and dose-dependently inhibited the growth of GV and AV, and L2 more potently inhibited growth than L1. When these samples were cultured in media, the amount of GV, AV, L1, or L2 in the medium was proportional to the qPCR data.

### 3.3. Inhibitory Effects of L1, L2, and PM on the Adhesion of GV to HeLa Cells

**Figure 6:**
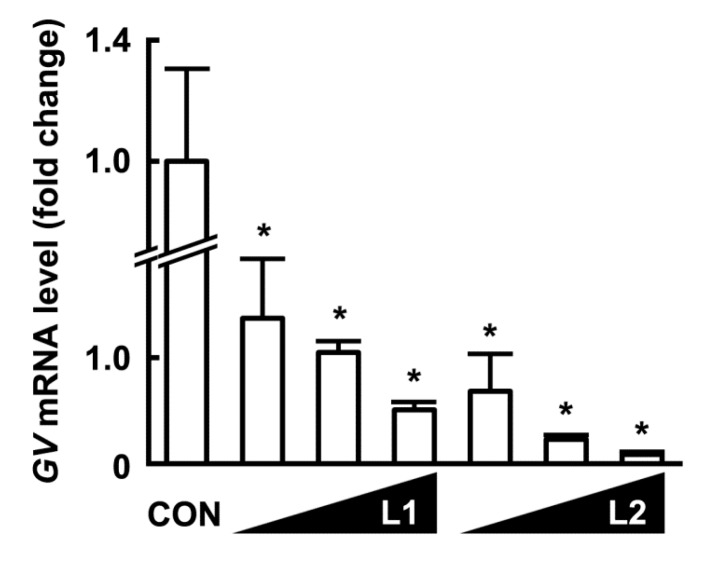
Effects of *Lactobacillus rhamnosus* HN001 (L1) and *Lactobacillus acidophilus* La14 (L2) on the adhesion of *Gardnerella vaginalis* (GV) to HeLa cells. GV (1 × 10^7^ CFU/mL) was infected in HeLa cells (1 × 10^7^ cells/mL), treated with probiotics (treated 1 × 10^5^, 1 × 10^6^, 1 × 10^7^ CFU/mL) 1 h after the infection of *G. vaginalis,* incubated at 37 °C in 10% CO_2_–90% air for 24 h, and then washed three times with saline. The numbers of *G. vaginalis* were assayed using qPCR. All data are expressed as mean ± S.D. (*n* = 4). * *p* < 0.05, vs. control treated with GV alone.

To understand the anti-BV mechanism of these probiotics, we first investigated the antagonistic effect of L1 and L2 on the adhesion of GV to a human cervical cell line (HeLa) in vitro (Figure 6). When HeLa cells were incubated with GV (1 × 10^7^ CFU), GV significantly adhered to the HeLa cells, demonstrated by qPCR. However, when HeLa cells were incubated with GV in the presence of L1 or L2, these probiotics significantly inhibited the adherence of GV to HeLa cells. Furthermore, L2 inhibited the adherence of GV more potently than L1. 

### 3.4. Effects of L1 and L2 on NF-κB Activation and TNF-a Expression in LPS-Stimulated Macrophages

**Figure 7:**
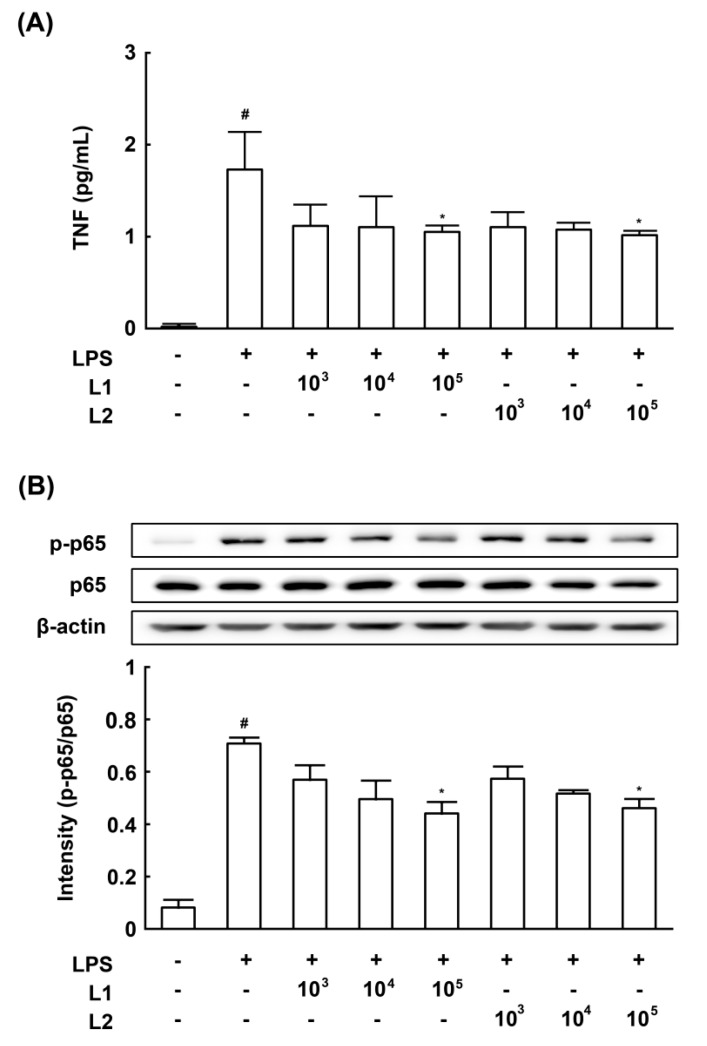
Effect of *Lactobacillus rhamnosus* HN001 (L1) and *Lactobacillus acidophilus* La14 (L2) on the expressions of TNF-α and the activation of NF-κB in LPS-stimulated murine peritoneal macrophages. Peritoneal macrophages (1 × 10^6^ cells) were treated with 50 ng/mL LPS in the absence or presence of L1 or L2 (1 × 10^3^, 1 × 10^4^, 1 × 10^5^ CFU/mL) for 90 min (for p65 and p-p65) or 20 h (for TNF-α). Normal control group was treated with vehicle alone. (**A**) Effect in NF-κB activation, measured by immunoblotting; (**B**) Effect in TNF-α expression, measured by ELISA. All data are expressed as mean ± SD (*n* = 4). ^#^ *p* < 0.05, significantly different vs. normal control group. * *p* < 0.05, vs. LPS control.

To determine whether the innate immune system is involved in the anti-BV mechanism of L1 and L2, we investigated the effect of L1 or L2 on NF-κB activation and TNF-α expression in LPS-stimulated macrophages (Figure 7). The results showed that L1 and L2 significantly inhibited LPS-induced NF-κB activation and TNF-α expression.

### 3.5. Effects of L1 and L2 on the Differentiation of Splenic T Cells into Th17 and Tregs Cells

**Figure 8:**
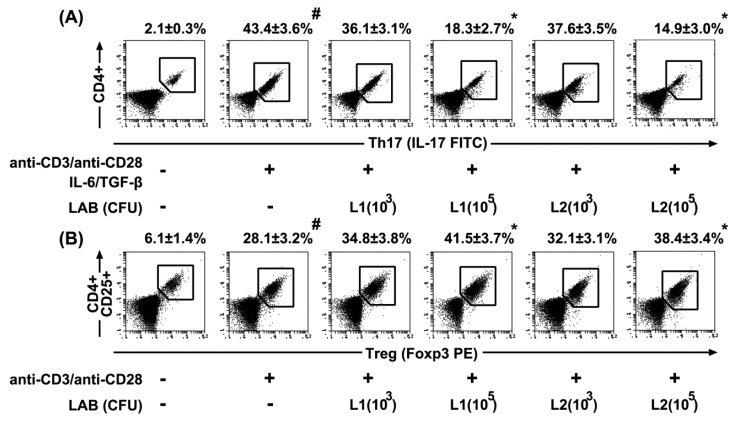
Effect of *Lactobacillus rhamnosus* HN001 (L1) and *Lactobacillus acidophilus* La14 (L2) on the differentiation of splenic Th cells into Th17 or Treg cells. (**A**) Effects on Th17 cell differentiation. Th17 cells were stained for cell surface CD4 and intracellular IL-17 and analyzed by flow cytometry; (**B**) Effects on Treg cell differentiation. Treg cells were stained for the cell surface CD4 and CD25 and intracellular Foxp3 and analyzed by flow cytometry. L1 or L2 (1 × 10^3^ or 1 × 10^5^ CFU/mL) were treated in splenic Th cells. All data are shown as the mean ± SD (*n* = 4). ^#^ *p* < 0.05 vs. normal control group. * *p* < 0.05 vs. control group treated without probiotics.

To determine whether L1 and L2 could regulate the differentiation of T cells involved in adaptive immunity, we investigated their effects on the differentiation of splenic Th cells into Th17 or Treg cells (Figure 8). Simulation of IL-6 and TGFβ in splenic CD4+ T cells with anti-CD3 and anti-CD28 antibodies significantly induced their differentiation into Th17 cells. However, treatment with L1 or L2 significantly suppressed the Th17 cell population, and L2 more potently inhibited Th17 differentiation than L1. Therefore, these probiotics induced T cell differentiation into Treg cells. In addition, these probiotics did not exhibit any cytotoxicity against splenocytes when treated for 5 days under the differentiation conditions.

## 4. Discussion

BV is an inflammatory vaginal disease caused by a disturbance of the vaginal microbiota, including a decrease in the number of beneficial bacteria, such as lactobacilli, and an increase in the number of harmful bacteria, such as GV and AV [4,10,21]. BV increases the risk of infection by sexually transmitted pathogens, such as HIV-1, and carries a risk of early delivery in pregnant women [22]. Although antibacterial drugs, such as clindamycin and metronidazole, are commonly used for BV treatment, probiotics are frequently recommended due to infection recurrence and drug resistance [7,8]. Unlike anti-bacterial drugs, microbiota-friendly probiotics may attenuate vaginosis by killing or out competing pathogens in the vagina [9,10,23].

In the present study, when L1 or L2 was orally administered to mice, these strains were detected in the vagina. In addition, when L1 and L2 were orally administered as a mixture (PM), both L1 and L2 were detected in the vagina, as previously reported [18]. Administration of L1, L2, or PM reduced the number of GV detected in the vagina. Moreover, these treatments reduced the adherence of GV on HeLa cells in vitro, as was previously reported for probiotics [24,25]. Both L1 and L2 produce hydrogen peroxide and lactic acid. We also found that these probiotics potently inhibited the growth of GV and AV in vitro, as was previously reported for other probiotics [26,27]. The numbers of these probiotic strains detected in the vagina following oral or intravaginal administration were not significantly different. However, oral administration more potently inhibited GV-induced myeloperoxidase activity, NF-κB activation, and TNF-α and IL-1β expression, which is involved in innate immunity, than intravaginal administration. Oral administration of L1, L2, or PM more potently inhibited GV-induced expression of RORγt, TNF-α, and IL-17, which are involved in adaptive immunity, when compared to the effects of vaginal administration. Furthermore, oral administration more potently increased GV-suppressed IL-10 and Foxp3 expression when compared to the increase following intravaginal administration. These results suggest that the anti-BV effect of orally administered L1, L2, or PM may be due to its regulatory effects on immune responses through the gastrointestinal tract rather than completion with or killing of GV in the vagina. These was supported by the reports that probiotics exhibited anti-inflammatory effects by inhibiting innate immune response, such as macrophage activation, by regulating NF-κB signaling, or by regulating adaptive immune response, such as Th cell differentiation [28,29]. In the present study, we found that these probiotics significantly inhibited NF-κB activation and TNF-α expression in LPS-stimulated macrophages, inhibited the differentiation of splenocytes into Th17 cells, and induced their differentiation into Tregs [28,30]. These results indicate that L1, L2, and PM can inhibit macrophage activation and regulate Th cell differentiation, suggesting that the anti-BV effect of L1, L2, and PM may be related to immune response regulation in the gut and vagina rather than direct competition or killing of GV in the vagina. Therefore, we conclude that L1, L2, and PM can attenuate GV-induced BV by regulating the innate and adaptive immune responses.
