# Correction: Jang, S.-E.; et al. *Lactobacillus rhamnosus* HN001 and *Lactobacillus acidophilus* La-14 Attenuate *Gardnerella vaginalis*-Infected Bacterial Vaginosis in Mice. *Nutrients* 2017, *9*, 531

**DOI:** 10.3390/nu9070715

**Published:** 2017-07-07

**Authors:** Se-Eun Jang, Jin-Ju Jeong, Su-Young Choi, Hyunji Kim, Myung Joo Han, Dong-Hyun Kim

**Affiliations:** 1 Department of Life and Nanopharmaceutical Sciences, College of pharmacy, Kyung Hee University, 26, Kyungheedae-ro, Dongdaemun-gu, Seoul 02447, Korea; jse3507@naver.com (S.-E.J.); bongoori7@naver.com (J.-J.J.); 2 Department of Food and Nutrition, Kyung Hee University, Seoul 02447, Korea; mjhan@khu.ac.kr; 3 NutriScience Co., Ltd, Seoul 06132, Korea; sychoifwp@nutribiotech.co.kr (S.-Y.C.); hyunji.kim@nutribiotech.co.kr (H.K.)

We would like to submit the following corrections to our recently published paper [1] due to the reported wrong name of probiotic mixture and the dose of some drugs. Moreover, new supplementary materials have been supplemented. The details are the following:

(1) “La-14” has been renamed to “GLa-14” throughout the paper.
(2) In the paragraph under Section 2.8, the dose of β-Estradiol-3-benzoate and suspension of GV have been corrected to 0.125 mg/30 μL and 1 × 10^8^ CFU /20 μL saline, respectively;
(3) In the Figure 1 legend, the dose of infected *G. vaginalis* has been corrected to 1 × 10^8^ CFU/mouse;
(4) In the paragraphs under Sections 3.2 and 3.3, sentences “PM also potently inhibited the growth of GV and AV (Figure S1)” and “PM also significantly inhibited the adherence of GV to Hela cells (Figure S2)” have been respectively supplemented;
(5) In the last sentence of Figure 5 legend, “AC” has been corrected to “AV”;
(6) Under Section 4, a new section, Supplementary Materials, has been supplemented.

These changes have no material impact on the conclusions of our paper. The manuscript will be updated and the original will remain online on the article webpage. We apologize for any inconvenience caused to our readers.
